# A new species of *Iotarphia* Cameron (Coleoptera, Staphylinidae, Aleocharinae) from Tasmanian seacoasts, Australia

**DOI:** 10.3897/zookeys.632.10657

**Published:** 2016-11-16

**Authors:** Seung-Gyu Lee, Andrew W Osborn, Kee-Jeong Ahn

**Affiliations:** 1 Division of Forest Biodiversity, Korea National Arboretum, Pocheon 11186, South Korea; 2 Honorary Research Associate, Queen Victoria Museum and Art Gallery, 2 Invermay Road, Launceston, Tasmania 7250, Australia; 3 Department of Biology, Chungnam National University, Daejeon 34134, South Korea

**Keywords:** Coleoptera, Staphylinidae, Aleocharinae, Athetini, Iotarphia, new species, Tasmania

## Abstract

*Iotarphia rufobrunnea* Lee & Ahn, **sp. n.** is described from Tasmania. The new species is compared with another species of the genus, *Iotarphia australis* Cameron. A description, habitus photograph and illustrations of the diagnostic characters are provided.

## Introduction

While working on aleocharine beetles collected by the second author from the eastern and southern seashores in Tasmania, Australia, we found specimens very similar to the athetine genus *Iotarphia* Cameron. After detailed examination of the specimens and comparison with *Iotarphia australis* Cameron (type species of *Iotarphia*), we concluded that these specimens represent a new species of the genus.

The athetine genus *Iotarphia* and its single described species have been recorded only in a “maritime habitat” from New South Wales and from Tasmania, both in Australia (Cameron 1943; Frank and Ahn 2011). Recently, Lee and Ahn (2015) synonymized the genus *Psammopora* Pace under *Iotarphia*. Little is known about their biology (Frank and Ahn 2011). In this paper, we provide a description, habitus photograph and line drawings of diagnostic characters of a new species of the genus *Iotarphia*.

## Method

Descriptive terms used here follow Sawada (1972), but we followed Ashe (1984) in some cases, particularly for mouthparts, to reduce confusion.

## Results

### Genus Iotarphia

Taxon classificationAnimaliaColeopteraStaphylinidae

Cameron, 1943

Iotarphia Cameron, 1943: 352. Type species: Iotarphia australis Cameron, 1943.
Psammopora Pace, 2003: 154. Type species: Psammopora delittlei Pace, 2003.

#### Diagnosis.

Members of *Iotarphia* are characterized by the combination of the following characters: labrum distinctly emarginate in anterior margin, with ε-sensillum conspicuously robust and blunt at apex; distal lobe of galea and lacinia developed, with many setae; ligula divided into two lobes; mentum emarginate in anterior margin; infraorbital carina absent; mesoventral process blunt at apex, reaching to half of mesocoxa; metaventral process narrow and pointed at apex; tarsal formula 4-5-5; metatarsi long (Pace 2003; Lee and Ahn 2015).

### Iotarphia rufobrunnea

Taxon classificationAnimaliaColeopteraStaphylinidae

Lee & Ahn sp. n.

http://zoobank.org/B2EABD60-7E6A-491D-834C-DD1520D42415

Figs 1 , 2–6 , 7–12

#### Material examined.

**Types. *Tasmania*.** Holotype, male (QVM:2014:12:0119), Coal Point, Bruny Island , collected 25.ix.2014, A.W. Osborn. Paratypes: 4, of which 3 (QVM:2016:12:1052 to 1054) share common collection data with holotype, and 1 (QVM:2014:12:0125) collected from Lighthouse Bay, Bruny Is., collected 24.ix.2014, A.W.Osborn.

All type specimens have been placed in the Queen Victoria Museum and Art Gallery, Launceston, Tasmania (QVMAG).

#### Description.

**Figure 1.**
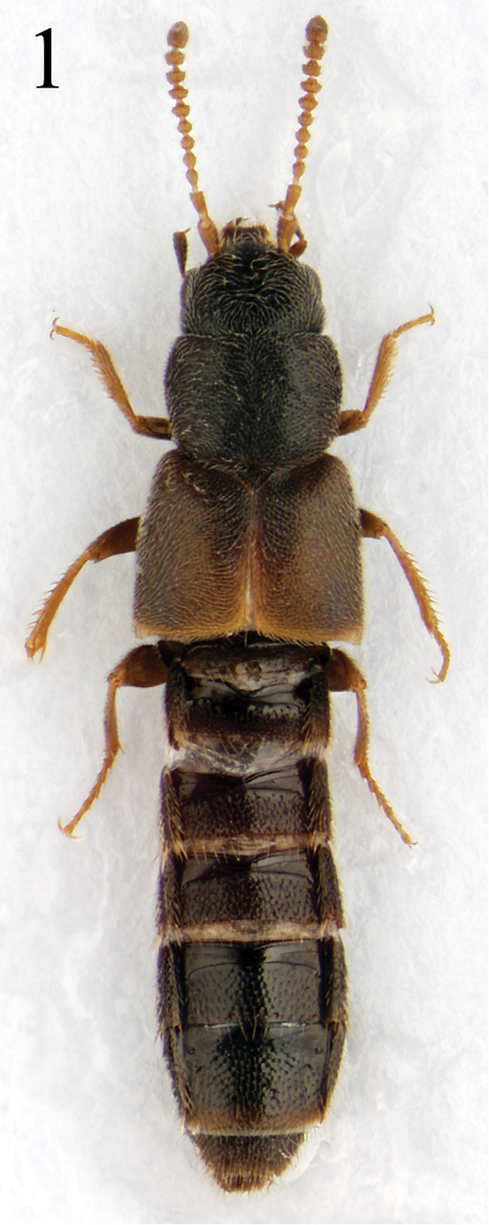
Habitus of *Iotarphia rufobrunnea* sp. n., 3.4 mm.

**Figures 2–6.**
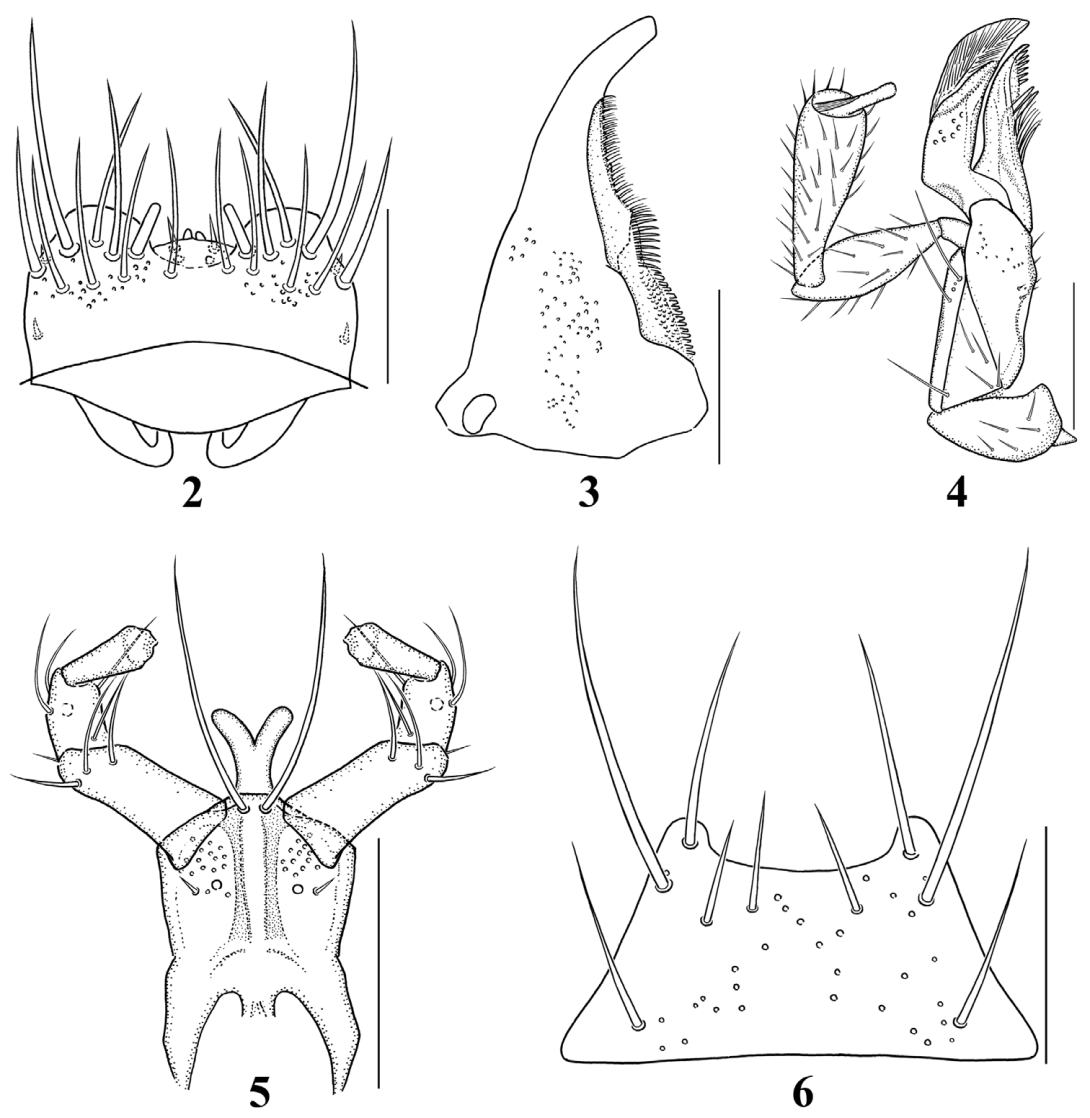
Mouthparts of *Iotarphia rufobrunnea* sp. n.: **2** labrum, dorsal aspect **3** right mandible, ventral aspect **4** right maxilla, ventral aspect **5** labium, ventral aspect **6** mentum, ventral aspect. Scale bars = 0.1 mm.

**Figures 7–12.**
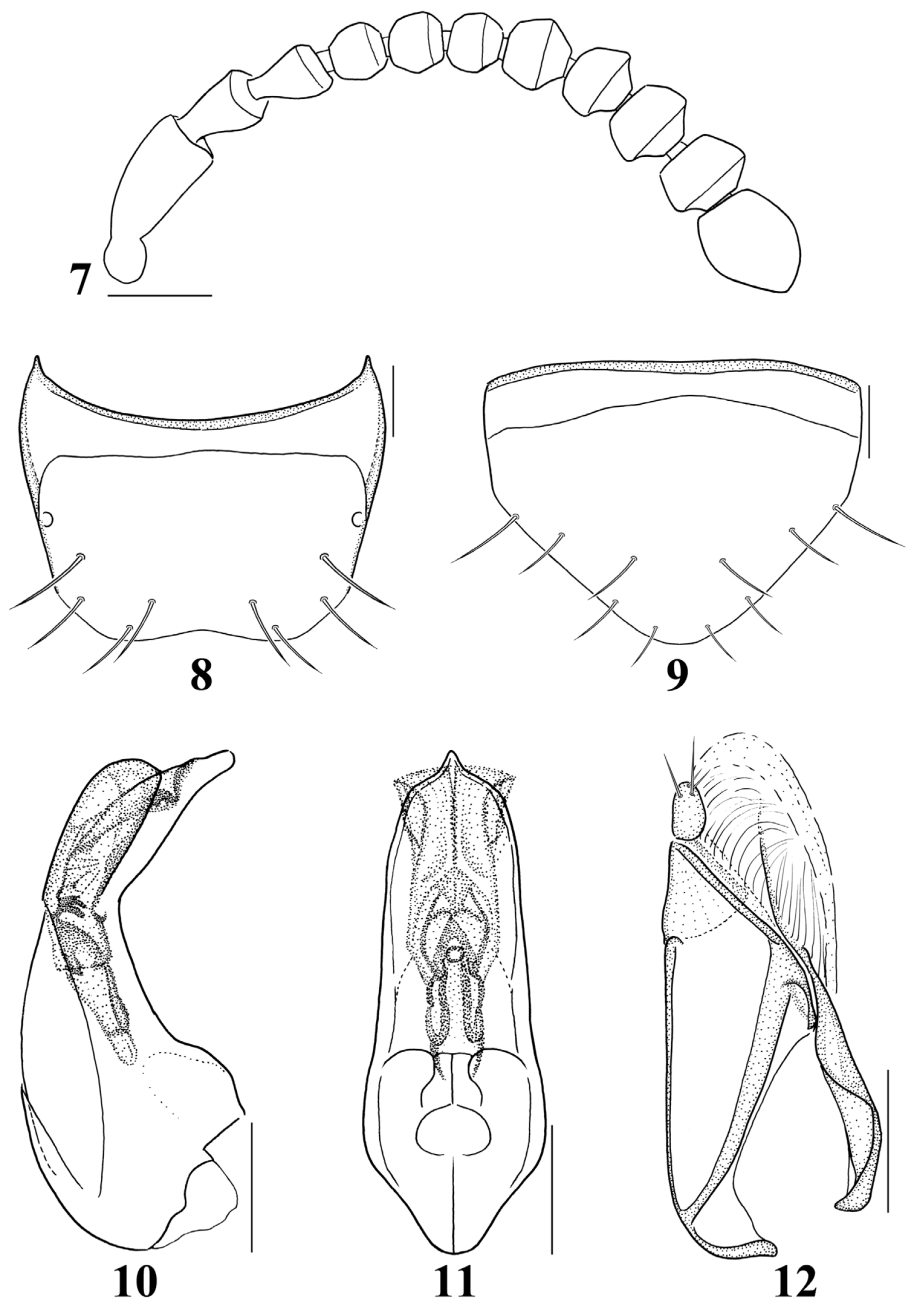
Diagnostic characters of *Iotarphia rufobrunnea* sp. n.: **7** antenna **8** male tergite VIII, dorsal aspect **9** male sternite VIII, ventral aspect **10** median lobe, lateral aspect **11** median lobe, ventral aspect **12** paramere, lateral aspect. Scale bars = 0.1 mm.

Length 2.8–3.5 mm. Body (Fig. 1) subparallel-sided and reddish brown to reddish black; head and abdomen almost black, antennae and legs reddish yellow, elytra reddish brown except for basal darker region; surface slightly glossy, densely pubescent with fine microsculpture. *Head*. Slightly transverse, approximately 1.1–1.2 times as wide as long, widest across eyes, narrower than pronotum; eyes slightly large and prominent, about 1.2 times as long as temples; gular sutures moderately separated, slightly diverged basally. Antennae (Fig. 7) slightly moniliform and about as long as head and pronotum combined; antennomeres 1–3 elongate, 1 longest, 2 distinctly longer than 3, 4–10 slightly to distinctly transverse, 11 longer than wide, slightly shorter preceding two combined. *Mouthparts*. Labrum (Fig. 2) with 8 macrosetae on each side of midline; epipharynx with several sensilla, including 2 lateral sensory rows on each side of midline; α-sensillum setaceous, about as long as ε-sensillum; β- and γ-sensilla short. Mandibles (Fig. 3) slightly asymmetrical, subtriangular, decurved and narrow apically, about 1.6 times as long as basal width, with blunt internal tooth; prostheca developed, composed of three portions, many small denticles present in molar region. Galea and lacinia of maxilla (Fig. 4) moderately long and slender; lacinia composited of seven small spines in distal comb region, two isolated spines longer; maxillary palpus distinctly 4-articled, elongate and pubescent; palpomere 1 smallest, 2 about 2.5 times as long as wide, 3 slightly longer than 2, about 3.0–3.2 times as long as wide, 4 digitiform and relatively short, filamentous sensilla reaching to basal half. Labium (Fig. 5) with ligula relatively broad and parallel-sided, divided into 2 lobes in basal half; medial pseudopore field of prementum very narrow, with several median pseudopores; two medial setae contiguous; two basal pores close together, one laterally behind the other; many lateral pseudopores, 1 setal pore and 1 real pore present on each side of midline; labial palpi 3-articled and elongate, with many setulae; palpomere 1 largest, about 2.0–2.5 times as long as wide, γ-setula slightly close to b-seta, 2 shortest, about 1.5–2.0 times as long as wide, 3 dilated apically and slightly shorter than 1, about 2.0–2.5 times as long as wide. Mentum (Fig. 6) trapezoidal, anterior margin distinctly emarginate. *Thorax*. Pronotum transverse, approximately 1.3 times as wide as long, widest at apical third; pubescence directed anteriorly in midline; hypomera fully visible in lateral aspect. Metanotal scutum with 1 long seta and about 3 short setae on each side of midline. Mesoventral process slightly longer than metaventral process, shorter than isthmus and metaventral process combined; isthmus slightly shorter than metaventral process. Metendosternite with distinctly elongate basal stalk and a pair of furcal arms. Elytra slightly longer and wider than pronotum; elytron approximately 1.6 times as long as wide, pubescence directed posteriorly and postero-laterally; postero-lateral margin almost straight; hind wings fully developed, flabellum composed of about 8 setose lobes. *Legs*. Moderately long and slender, with dense pubescence and macrosetae; pro- and mesotibiae with small and blunt spines along outer surface; length ratio of tarsomeres 36:38:40:78 (protarsus); 40:43:46:48:78 (mesotarsus); 48:55:58:54:80 (metatarsus); one empodial seta present, shorter than claw. *Abdomen*. Subparallel-sided; surface glossy and densely pubescent, with transverse and imbricate microsculpture; male tergite VIII (Fig. 8) with 4 macrosetae on each side of midline, posterior margin slightly emarginate; male sternite VIII (Fig. 9) with about 5 macrosetae, posterior margin convex, long marginal setae present; *Aedeagus*. Median lobe (Figs 10–11) elongate oval, apical process elongate and convergent apically in ventral aspect, and slightly bent in lateral aspect. Apical lobe of paramerites (Fig. 12) narrow apically, with four setae; a-seta longest, b-seta longer than c-seta, d-seta shortest and close to c-seta and positioned at apex.

#### Etymology.

Named from the Latin *rufobrunnea* meaning “reddish brown”, which refers to the elytra color.

#### Distribution.

**Figure 13.**
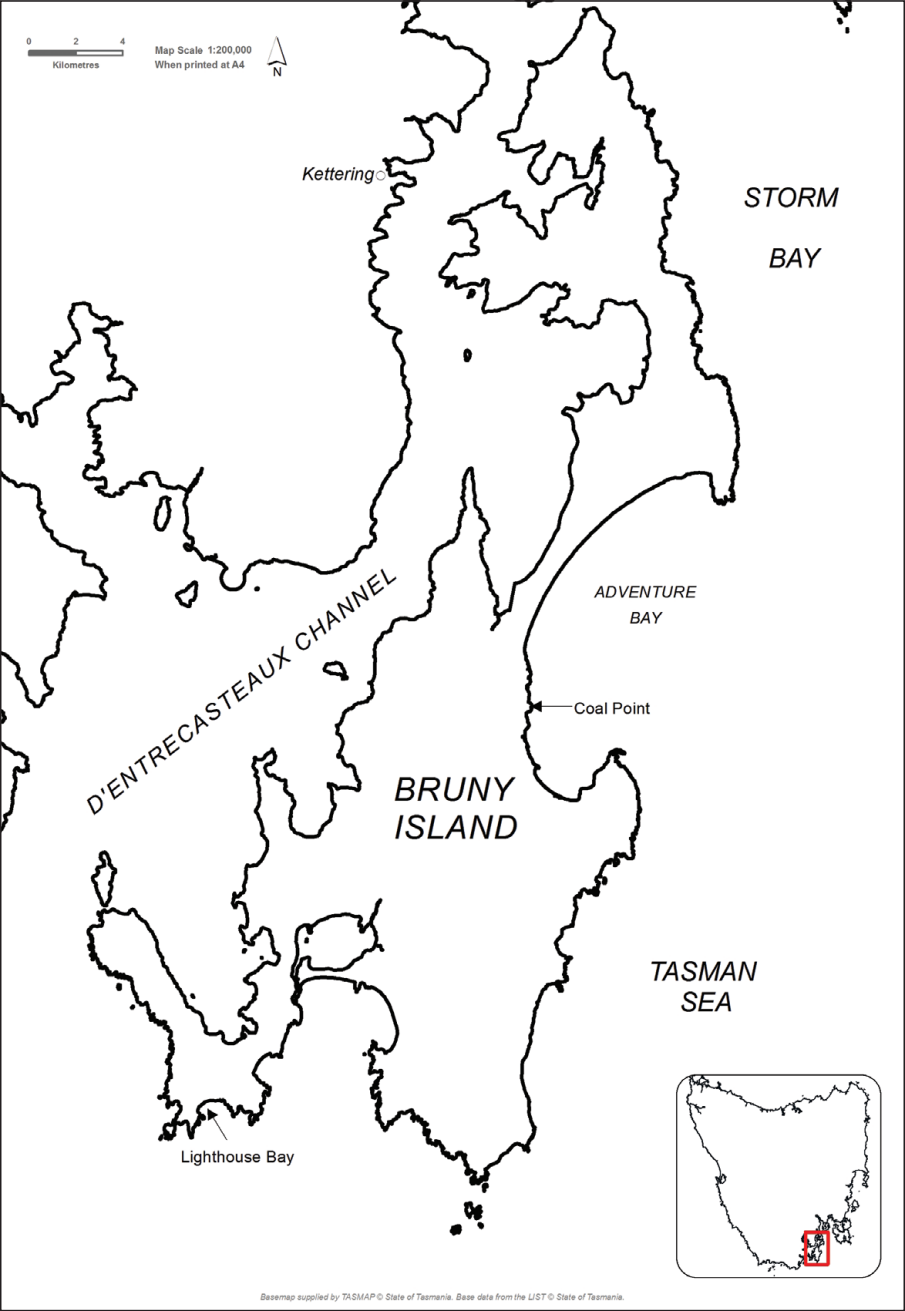
Distribution map.

Bruny Island, at both Lighthouse Bay and Coal Point (refer to map below), Tasmania, Australia (Fig. 13).

#### Remarks.

This species is similar to *Iotarphia australis*, but can be distinguished by the characters provided in Table 1 and the shape and structure of the aedeagus. The specimens of the new species were collected on Bruny Island from (i) an entirely sandy substrate just into the supra-littoral zone at Coal Point (geographical coordinates: 43.34211°S and 147.32178°E) and (ii) from a sandy substrate in which some small rocks were present within the littoral zone at Lighthouse Bay (geographical coordinates: 43.48616°S and 147.15022°E).

**Table 1.** Differences between *Iotarphia australis* Cameron and *Iotarphia rufobrunnea* sp. n.

	*Iotarphia australis*	*Iotarphia rufobrunnea* sp. n.
Length	2.2–3.0 mm	2.8–3.5 mm
Elytra color	yellow in most regions	reddish brown
Antennomere 4	about as wide as long	slightly transverse
Mesoventral process	shorter than metaventral process	longer than metaventral process
Meso- and metaventral processes	contiguous	separated

The description of the new species within the present paper brings the total number of coastal Staphylinidae species in the Tasmanian fauna to five: *Iotarphia australis* (= *Psammopora delittlei* Pace), *Iotarphia rufobrunnea* Lee & Ahn, sp. n., *Teropalpus pictipes* (Lea), *Cafius pacificus* (Erichson), and *Remus sericeus* (Holme).

## Supplementary Material

XML Treatment for Iotarphia

XML Treatment for Iotarphia rufobrunnea
